# Book Notices

**Published:** 1894-06

**Authors:** 


### Book Notices.

THE PRISON QUESTION. By Charles H. Reeve, of Plymouth, Indiana. A theoratical and philosophical review of some matters relating to Crime, Punishment, Prisons, and Reformation of Convicts; with a glance at Mental, Social and Political Conditions, and some suggestions about Causes, the Prevention of Crime and of the Production of Criminals. Designed to show how society may protect itself against the disorderly element and check the rapid increase in the prison population. Copyright. Octavo, 200 pages, printed on heavy tinted and calendered paper, in clear, handsome type, leaded, widely spaced, and neatly bound in best cloth. Sent by mail on receipt of price—$1.25. Address the author, at Plymouth, Indiana.

There is no subject which more vitally affects the interests of the general public and which more imperatively demands attention, consideration, and judicious action, on the part of all the moral and intelligent people of the nation, than does the one relating to the defective and criminal portions of the population.

The aim of this book is to call attention to the subject, and in an order not before attempted, to present some facts as to some of the conditions, causes and relations involved in it, with suggestions for improvements in the methods of dealing with them.

"I can hardly conceive of a work which could condense into so small a space as lucid as scientific an exposition of the highest and latest development of the subject. . . . I have nowhere seen so bold, fearless and truthful expression of the leading features of the prison problem, and I firmly believe that, if it is worked out on the lines laid down in this treatise, more tangible results will follow in the diminution of the volume of crime than has been attained during all the years of discussion."---*Captain John W. Pope, U. S. Army Commandant of the Military Prison, Leavenworth, Kans.*

We have read the book with great interest, and can commend it to our readers as the best book on the subject we have ever seen.

---

A Manual of Nursing in Pelvic Surgery. By Lewis S. McMurtry, A. M., M. D., Professor of Gynecology in the Hospital College of Medicine, Louisville; Surgeon-in-Charge of the Jennie Casseday Infirmary for Women; Gynecologist to Sts. Mary and Elizabeth Hospital, etc. 82 pages. Illustrated. John P.Morton & Co., Publishers, Louisville, Ky.

This book is designed especially for nurses, but contains many points that may be studied with benefit by the surgeon. The first chapter, on "General Considerations," gives instructions to the nurse on the pelvic, the pelvis organs and their diseases; and the operations necessary for the relief or cure of these diseases. The second chapter treats more particularly of surgical cleanliness, and the necessity for its observance and enforcement by the nurse. Then follow chapters on each of the following subjects: Preparation of instruments, etc., Preparation of patient, The care of the patient after the operation, Complications, Operations in private houses.

It is a valuable little book, and Dr. McMurtry has done the profession a service in getting it up in a form so convenient and practical for the nurse; and every surgeon should see to it that those who are to nurse his gynecological cases are provided with a copy of it. H.

---

Operative Surgery. By Th. Kocher, M. D., Professor at the University and Director of the Surgical Clinic at the Berne University. 8vo, 288 pages, 163 illustrations. Extra muslin, price, $3.00. William Wood and Company, Publishers, 43, 45, 47 East Tenth St., New York City.

To become a successful surgeon it is absolutely necessary that the operative technique should be mastered. This is the branch of surgical science in which most young surgeons are deficient in knowledge, and in which the majority of our surgical text-books are sadly wanting. It is the purpose of the author of this book to provide a concise and ready hand-book, giving the different steps of the various surgical operations, and by means of which the operator may quickly post himself. The author is very explicit in his instructions for making the first incision, especially its direction and depth.

Besides the consideration of the actual operations, the book gives full directions for the preparation of the patient, the operating room, surgical dressings, etc. Anæsthetics are also given careful consideration, and their relative advantages and disadvantages are discussed.

The book is printed on excellent paper, and is handsomely bound. It will meet with appreciation and will fill an important place in the library. H.

---

INTERNATIONAL CLINICS.—A Quarterly of Clinical Lectures on Medicine, Neurology, Pædiatrics, Surgery, Genito-Urinary Surgery, Gynecology, Obstetrics, Ophthalmology, Laryngology, Otology, and Dermatology. By Professors and Lecturers in the leading Medical Colleges of the United States, France, Great Britain, and Canada. Edited by Judson Daland, M. D., Philadelphia, Instructor in Clinical Medicine and Lecturer on Physical Diagnosis in the University of Pennsylvania, etc., etc. J. Mitchell Bruce, M. D., F. R. C. P., London, England, Physician and Lecturer on Therapeutics at the Charing Cross Hospital. David W. Finlay, M. D., F. R. C. P., Aberdeen, Scotland, Professor of Practice of Medicine in the University of Aberdeen; Physician to, and Lecturer on Clinical Medicine in the Aberdeen Royal Infirmary, etc. Volume I, Fourth series, 358 pages octavo. Illustrated. Sold only by subscription. Price per volume, cloth, $2.75; half leather, $3.00. Price per series, (4 volumes), cloth, $11.00; half leather, $12.00. J. B. Lippincott Co., publishers, Philadelphia.

This work is a collection of the clinical lectures delivered by leading medical teachers in this country, Great Britain and Canada, as reported by competent medical stenographers, and each lecture is afterwards revised by the professor or lecturer who delivered it. Only those lectures are selected which are of the most practical interest to the physican, and they are grouped and arranged by the editors in the form best suited to a work of this character.

It is not at all surprising that the "Clinics" is growing rapidly in popularity, as it furnishes the nearest approach to an attendance on a post-graduate course, once every three months. Each article is from an experienced teacher, and represents the very latest views of the best men in the medical profession. The "Clinics" is free from antiquated and out-of-date matter, and we are more pleased with it now than ever before. H.

MEDICAL JURISPRUDENCE, FORENSIC MEDICINE, AND TOXICOLOGY. By R. A. Witthaus, A. M., M. D., Professor of Chemistry, Physics, and Hygiene, in the University of the City of New York, etc., etc., and Tracy C. Becker, A. B., LL. B., Counselor-at-Law and Professor of Criminal and Medical Jurisprudence in the University of Buffalo. In four volumes. Volume I. Large 8vo, 845 pages, illustrated with wood-cuts and two lithographic plates in colors. Price, in muslin, $5.00; in brown sheep and in law style, $6.00 per volume. Sold by subscription only. William Wood and Company, Publishers, 43, 45 and 47 East Tenth St., New York.

In the preparation of this work the editors have followed the same method as that which was used in the preparation of "The Reference Handbook of the Medical Sciences," i. e., to each contributor has been assigned that subject to which he has devoted most study and research. The list of contributors has been carefully selected from among the leading men of both the medical and legal professions, and it may be said to the credit of those who have contributed to volume I. that the excellence of their contributions, severally and collectively, demonstrates the wisdom of the selections made by the editors. The following is the list of contributors tot his volume:

T. C. Becker, Esq., Chas. A. Boston, Esq., Hon. Wm. A. Poste, August Becker, Esq., H. P. Loomis, M. D., J. C. Rosse, M. D., George Woolsey, M. D., Roswell Park, M. D., E. V. Stoddard, M. D., W. N. Bullard, M. D., D. S. Lamb, M. D. The introduction, comprising twenty-five pages, and giving a short history of medicine and jurisprudence, together with medical and medico-legal writings, from the earliest periods of medical history up to the present time, was written by Dr. R. A. Witthaus, one of the editors.

In the arrangement of the matter, the primary divisions into the three sciences of medical jurisprudence, forensic medicine, and toxicology, has been adopted. In this volume the division of pure medical jurisprudence, and the thanatological division of forensic medicine are considered. The application of the microscope to forensic medicine, and the bio-thanatological and biological divisions of the same will be treated of in the second and third volumes, while the division of toxicology will be contained in the fourth volume.

It is safe to say that if the succeeeding volumes of this work come up to the high standard of excellence as established by volume I, it will be many years before there is a work on medical jurisprudence to compare with it. It is by far the best work on the subject yet published. H.

THE TREATMENT OF CUTANEOUS MALIGNANT EPITHELIOMATA (CANCERS). By A. R. Robinson, M. B., L. R. C. and S. (Edin.), Professor of Dermatology at the New York Polyclinic; Attending Physician to the New York Cancer Hospital; Member of the American Dermatological Association, etc., etc., 63 pages. Illustrated. Price, cloth, $1.00 The International Journal of Surgery Co., Publishers, 14 Platt St., New York City.

The etiology of cancer being a much disputed question, the author of this little volume had the good judgment to have but little to say on this question. He has confined his remarks principally to the pathology and treatment of this variety of cancer. In the matter of treatment he reviews the different surgical procedures, giving the advantages of each method, and he seems to favor in almost all cases, either the excision method or the use of caustics in the form of pastes. In comparing these two methods, he says: "The use of certain caustic agents, judiciously and properly applied, is of the greatest service in the treatment of cutaneous cancers, and, in the majority of cases, far superior to the knife in securing their permanent removal with the least amount of deformity."

We are highly pleased with the book, and we do not hesitate to say that every physician in this country should be the possessor of a copy of it. Many cases of epithelial cancer can be readily and easily cured if an appropriate line of treatment is adopted in the beginning, and no practicing physican can afford not to be in possession of the necessary information. H.

---

HOW TO USE THE FORCEPS: With an introductory account of the Female Pelvis and of the Mechanism of Delivery. By Henry G. Landis, A. M., M. D., Professor of Obstetrics and Diseases of Women and Children in Starling Medical College, Columbus, O. Revised and enlarged by Charles H. Bushong, M. D., Assistant Gynecologist to Demilt Dispensary, New York. 203 pages; 27 illustrations. Price, cloth, $1.75. E. B. Treat, Publisher, 5 Cooper Union, New York City. 1894.

No one should attempt to practice obstetrics until he is perfectly familiar with the use of the forceps. Yet practitioners are often found who are not only ignorant of the anatomy of the pelvic organs, the mechanism of delivery and the indications for the use of the forceps, but who are actually unable to introduce the forceps, and totally ignorant of the proper way to make traction. To all such we would recommend this book by Drs. Landis and Bushong. In Part I. is discussed the *Mechanism of Labor*, the anatomy of the pelvis, the propelling forces, the body to be propelled, the mechanism of delivery, and the different presentations, positions, etc. Part II. is devoted to the consideration of the forceps, the different makes and shapes. *The application of the forceps*,—at the inlet, at the outlet; on the after-coming head—traction, compression, leverage, flexion, and rotation. *When to use the forceps*,—during the second stage, during the first stage, for certain accidents of labor, and for secondary purposes. Part III.—*Application and Cases.* Critical remarks, the perineal body, the use of the forceps, preparation for their use, symphyseotomy, and illustrated cases. H.

---

AN AMERICAN TEXT-BOOK OF GYNECOLOGY, MEDICAL AND SURGICAL, FOR STUDENTS AND PRACTITIONERS. By Henry T. Byford, M. D.; J. M. Baldy, M. D.; Edwin B. Cragin, M. D.; J. H. Etheridge, M. D.; William Goodell, M. D.; Howard A. Kelly, M. D.; Florian A. Krug, M. D.; E. E. Montgomery, M. D.; William R. Pryor, M. D.; George M. Tuttle, M. D. Edited by J. M. Baldy, M. D. In one handsome royal octavo volume of 713 pages, with 360 illustrations in text, and 37 colored and half-tone plates. Price, cloth, $6; sheep, $7; half Russia, $8. W. B. Saunders, Publisher, 925 Walnut Street, Philadelphia.

Saunders' text-books are becoming very popular, and they deserve to be more widely known and more generally studied by both students and practitioners. This new candidate for honors is prepared on the same plan of the American Text-Book of Surgery. It is the product of the combined labors of a number of our best gynecologists, all of the authors being teachers. The subjects are discussed from the standpoint of a teacher, and the large number and fine quality of the illustrations enable the authors to make themselves readily understood without going into explanatory details. It is largely a system of object lessons, being at once short and comprehensive, and they will for this reason be longer remembered.

In the book, the various subjects belonging to gynecology are ably discussed, and it is free from the extremes usually met with in a book all of which is written by one man. It is true that some new subjects have been introduced, and a number of old ones have been discussed from an entirely new standpoint, but the many advances that have recently been made in this branch of medical and surgical science necessitate these changes. Indeed, it is largely for this purpose that the book has been written. A medical book at this time is almost worthless if it is not "up to date."

Dr. Baldy is to be commended for his excellent work, the securing of the services of his eminent assistants, and the good fortune of having so enterprising a publisher.

The book will no doubt take first rank among the works on this subject. H.

---

THE INTERNATIONAL MEDICAL ANNUAL AND PRACTITIONERS' INDEX: A Work of Reference for Medical Practitioners. The conjoint authorship of thirty-nine distinguished American, British and Continental authorities. 703 pages, 29 plates, and 70 wood cuts. Price, cloth, $2.75. E. B. Treat, Publisher, 5 Cooper Union, New York. 1894.

This is the twelfth year of publication of the International Medical Annual, and the present volume—1894—is larger and better than any of its predecessors. It contains a concise, and yet complete report of the progress of medical science, in all of its branches, throughout the world. As a practical ready reference work, it has no equal. The design of the book is to bring the general practitioner into communication with those who are advancing the science of medicine, and to furnish him with a concise report of all that is new and worthy of preservation. With this book in his possession, the doctor will find it comparatively easy to keep up with the advanced thinkers of the profession, and the low price at which the publisher is offering it places it within the reach of all. H.

---

OUTLINES OF PRACTICAL HYGIENE ADAPTED TO AMERICAN CONDITIONS. By C. Gilman Currier, M. D., Visiting Physician to the New York City Hospitals; Fellow of the New York Academy of Medicine; Member of the New York Pathological Society; Member of the American Medical Association, etc. One large octavo volume, 468 pages. Illustrated. Price, cloth, $2.75. E. B. Treat, Publisher, 5 Cooper Union, New York.

The author of this book is a practical sanitarian and an expert specialist; and recognizing, as he says, "That 'Preventive Medicine' is not only the 'medicine of the future,' but is fast becoming the 'medicine of to-day,' and that it is the duty of every physician to utilize all the known means for the prevention of disease and the prolonging of human life," he undertook the preparation of this volume. To assist him in its preparation, he had access to the principal books, monographs, periodicals, etc., bearing on this subject, and the assistance of a number of the leading scientific men of this country. The purpose of the author was to elucidate the truths of science, and not to mask them. He has endeavored to use the simplest language possible to convey his ideas, and to eliminate, as far as possible, Greek and other foreign synonyms. The book is a practical one, and possesses unusual merit. The following list of contents, from chapter headings, will give some idea of the scope of the work: Soil; Climate; Protection of Body; Clothing; Bathing; Personal Hygiene; Physical Exercises; Schools; Occupations—Their Influence on Health; Heating; Lighting; Buildings; Ventilation; Diet; Foods—Their Preparation and Adaptation; Water and Water Supplies; Fluid Waste; Sewers; Drainage; Plumbing; Garbage and Other Refuse; Disposal of the Dead; Human Excreta, Disposal of; Bacteria and Disease; Infectious Diseases; Disinfection; Restriction; Communicable Diseases. H.

---

Outlines of Obstetrics: A Syllabus of Lectures Delivered at the Long Island College Hospital. By Charles Jewett, A. M., M. D., Professor of Obstetrics and Pædiatrics in the College, and Obstetrician to the Hospital. Edited by Harold F. Jewett, M. D. 264 pages. Price, in cloth, $2. W. B. Saunders, Publisher, 925 Walnut St., Philadelphia.

This syllabus is prepared for the especial use of the medical student, to assist him in the didactic and practical work during his college course. The author's idea is, and it is a correct one, that if the student has a good foundation upon which to build his superstructure, if he is well grounded in the general facts and principles. secures a classified knowledge of the outlines of his subject, the acquisition of a complete and systematic knowledge of the subject becomes a matter of comparatively easy growth. Perhaps no one can more fully appreciate the work of the author of this volume, than the medical student, who realizes that he has not the time to read over the average medical text-book, much less remember what it contains. It is very evident that the student would learn much more from a short treatise, giving all the principal and practical facts, than from a voluminous work. This book will also be found an excellent hand-book for ready reference by the busy practitioner. It contains many hints and practical points not found in the ordinary text-book. H.

---

The Journal is in receipt of the new sixth edition of A. S. Aloe Company's Surgical Instrument Catalogue. This is a nice volume, of nearly eleven hundred pages, bound in cloth, with rich, gold letters, and profusely illustrated throughout. It contains not only an illustration of the instrument, but a description of its use and the operations in which it is needed. The old plan of quoting gross prices and furnishing a discount sheet, has been abandoned, and the much better plan of printing net prices for each and every instrument has been adopted. This will save much time and labor to the purchaser. This magnificent catalogue is a necessity in the surgeon's office, and it can be had for the small sum of 50 cents, to pay express charges.

